# Exploring the Possible Link Between Diabetic Ketoacidosis, Glucose-6-Phosphate Dehydrogenase Deficiency, and Methemoglobinemia

**DOI:** 10.7759/cureus.61656

**Published:** 2024-06-04

**Authors:** Hussin A Alsubaie, Ghadeer A Alsubaie, Ahmed M Almusalami

**Affiliations:** 1 Internal Medicine, King Fahad General Hospital, Al-Ahsa, SAU; 2 Medicine, King Faisal University, Al-Ahsa, SAU

**Keywords:** anemia, hemolysis, methemoglobinemia, hemoglobin, diabetic ketoacidosis (dka), glucose-6-phosphate dehydrogenase (g6pd)

## Abstract

One of the most widespread enzymopathies affecting human beings is glucose-6-phosphate dehydrogenase (G6PD) deficiency, which is brought on by inherited mutations in the X-linked gene. Red blood cells (RBCs) with a G6PD deficiency are more sensitive to oxidative assault and consequently to hemolysis. There are more than 200 known G6PD mutations, of which around half are polymorphic and thus prevalent in a variety of populations. We present a case of diabetic ketoacidosis (DKA), with severe hemolytic anemia and methemoglobinemia. The patient was admitted to the intensive care unit, treated for DKA, and received a blood transfusion. In addition, the patient presented with high methemoglobin levels and features of severe hemolytic anemia from the onset, which made the diagnostic consideration of G6PD highly likely. Accordingly, the patient was treated with several doses of ascorbic acid instead of methylene blue. In a nutshell, a patient with DKA who has hemolytic anemia has to have it properly evaluated and controlled. The link between methemoglobinemia, G6PD deficiency, and DKA should be recognized by medical professionals, particularly when oxygen saturation gaps are found.

## Introduction

Glucose-6-phosphate dehydrogenase (G6PD) deficiency is an inherited hemolytic disorder caused by a defect in the X chromosome, which is characterized by low G6PD enzyme levels in the red blood cells (RBCs) that produce nicotinamide adenine dinucleotide phosphate oxidase (NADPH) to protect the RBCs from oxidative injuries, preventing it from premature hemolysis [1]. Around 400 million people are affected with G6PD deficiency globally, mainly in Africa, Asia, the Middle East, the Mediterranean, and Latin America, making the disease the most prevalent enzymatic disorder worldwide [2]. The prevalence of G6PD deficiency in Saudi Arabia was 4.76%, which increased drastically parallel with the initiation of neonatal screening in the Al-Ahsa and Al Qatif regions, accounting for 14.7% and 30.6%, respectively, in one year [3]. According to the World Health Organization (WHO), there are five different classes of G6PD deficiency. Class I is characterized by chronic hemolysis without the need for the presence of a stressor. Class II is defined as having an enzyme activity of less than 10%; an enzymatic activity of 10%-60% is considered moderate enzyme activity and referred to as class III. Class IV is considered when there is normal enzyme activity of 60%-150%, and lastly, class V is defined as increased enzyme activity of more than 150% [4]. There are three common triggers of hemolysis in patients with G6PD deficiency; this includes infections, drugs, and foods; among these, infection is the most commonly cited trigger, as well as some drugs, such as antimalarial medication and sulfa-containing drugs, and certain foods, including legumes and fava beans [5]. Furthermore, recent studies have shown that increased blood sugar levels such as in diabetic ketoacidosis (DKA) conditions can further impair the function of the G6PD enzyme, causing further cell damage and hemolysis [6]. G6PD deficiency patients usually remain asymptomatic until they are exposed to one of its triggers; then, the patients present with hemolytic anemia symptoms such as pallor, jaundice, and easy fatigability [7]. Methemoglobinemia is a syndrome that results in the suppression of the reduction of methemoglobin to hemoglobin and so failure to bind to oxygen. This can occur in rare cases among individuals with G6PD deficiency, and it causes the residual oxyhemoglobin to have a larger affinity for oxygen, which causes tissue hypoxia [8].

## Case presentation

This is a case report of a 35-year-old male who worked as a cashier in a supermarket. He had been a known case of diabetes mellitus for eight years. The patient was on metformin and sitagliptin and had poor compliance with his medications for the last eight months. He presented to the emergency department with anemia symptoms in the form of dizziness, exertional dyspnea, palpitation, generalized body pain, and easy fatigability for four days. He had nausea, vomiting, generalized abdominal pain, and dark tea-colored urine for two days.

However, he did not notice any change in his eye color. He has a family history of G6PD in his cousin. He has no previous episodes of DKA or any medical admission. There is no family history of methemoglobinemia. In fact, he reported consuming food items that are known to trigger bouts of hemolysis in subjects with G6PD in the past without any apparent consequence. He denies recent intake of medication including herbal and states that there is no clear exposure to chemicals, benzene, radiation, or certain odors.

On physical examination, he had pallor and deep jaundice. The vitals were remarkable for tachycardia of 110 beats per minute and tachypnea of 26 breaths per minute with an oxygen saturation (SpO_2_) of 77% at room air, which reached 82% on 15 L of O_2_ (Table 1). There was no fever, and he was able to maintain normal blood pressure. The examination of other systems was unremarkable, and he was alert and well oriented to time, place, and person.

**Table 1:** Vital signs SpO_2_: oxygen saturation

Vital signs	Initial	After five days	Reference range
Oxygen saturation	SpO_2_ of 77% on room air and 82% on 15 L of O_2_	98% on room air	95%-100%
Random blood sugar	23.6 mmol/L	7.7 mmol/L	3.9-7.8 mmol/L
Blood pressure	110/78 mmHg	125/80 mmHg	90-120/60-80 mmHg
Pulse rate	110 beats per minute	98 beats per minute	60-100 beats per minute
Respiratory rate	26 breaths per minute	18 breaths per minute	12-18 breaths per minute
Temperature	36.8°C	36.5°C	36.5-37.3°C

**Figure 1:**
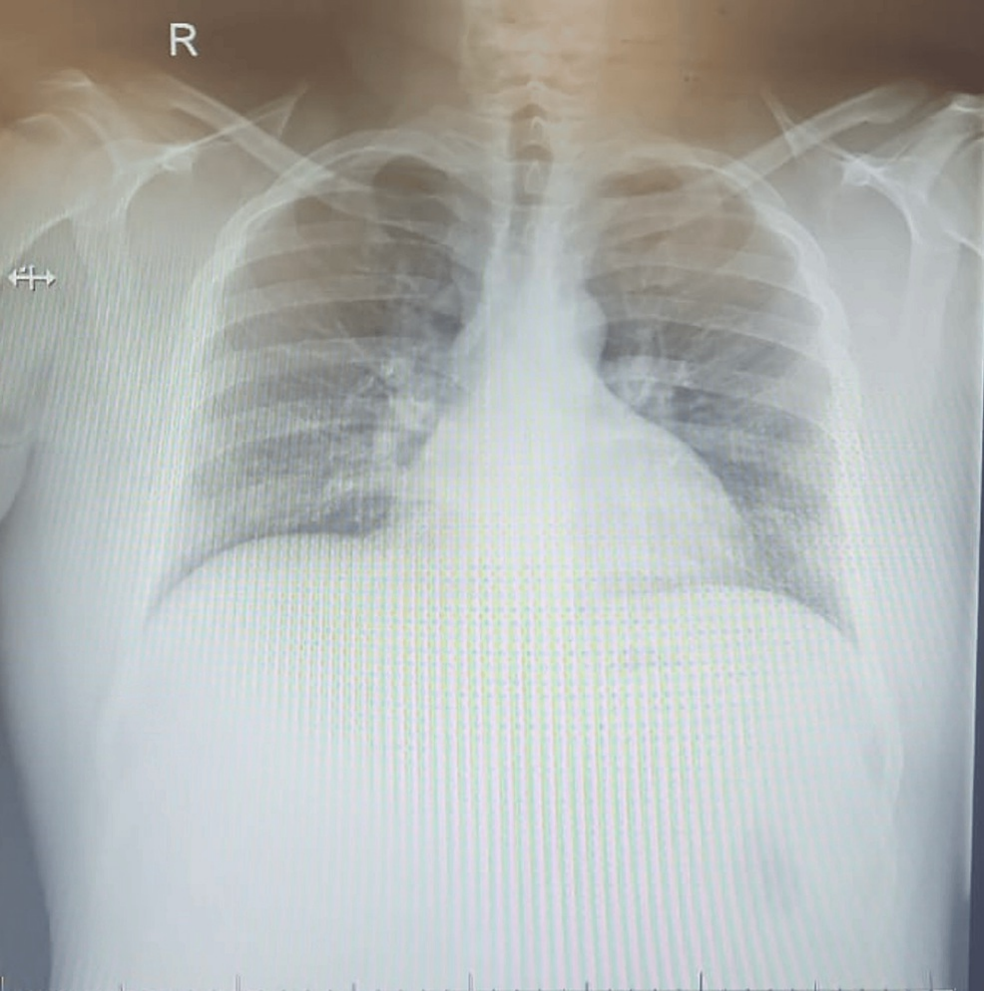
Chest X-ray

Investigations show that his random blood sugar (RBS) was 23.6 mmol/L on arrival, and the dark urine was positive for ketone and glucose; further details are mentioned in Table 2. The arterial blood gas (ABG) results revealed oxygen saturation of arterial blood (SaO_2_) of 94%, which is within normal limits despite the requirement of high-flow O_2_ for his clinical desaturation. There was a clear discrepancy between the two oxygen saturation measurements. The methemoglobin level of 14.1% was detected by the arterial blood gas test (ABG) (Table 3). The white blood cell count was 28.4 × 10^9^/L, which is high. A septic workup was done and showed no evidence of an infection that might explain the cause of leukocytosis. The blood culture did not show any growth. A chest X-ray has been done and showed no signs suggestive of infection or any other abnormalities, as shown in Figure 1. Based on that, we believe that leukocytosis was related to dehydration and hormonal stress due to his condition. The hemoglobin dropped to 8.5 g/dL from his baseline of 14 g/dL with a significant increase in the hemolytic markers (Table 4). The workup for other hemolytic conditions such as hemoglobinopathies and autoimmune conditions, which are common in our setup, was none revealing (Table 5). A peripheral blood smear was done; the findings are demonstrated in Table 6.

**Table 2:** Urine analysis

Test	Result	Reference range
Urine appearance	Clear, light orange	Yellow
Glucose	+4	Negative
Ketone	+3	Negative
Blood	+4	Negative
Urine red blood cell	0-1 per high-power field	0-4 per high-power field
Urine white blood cell	0-2 per high-power field	0-5 per high-power field
Microorganism or yeast	None	None
Nitrate	Negative	Negative
Urine bilirubin	Negative	Negative

**Table 3:** Initial arterial blood gas (ABG) PaCO_2_, partial pressure of carbon dioxide; PaO_2_, partial pressure of oxygen

Test result	Value	Reference range
pH	7.29	7.35-7.45
PaCO_2_	31 mmHg	35-45 mmHg
PaO_2_	94 mmHg (on oxygen)	80.0-108.0 mmHg
Bicarbonate (HCO_3_)	15.9 mmol/L	22.0-26.0 mmol/L
Methemoglobin	14.1%	0.0%-1.5%

**Table 4:** Summary of the laboratory test results

Test result	Initial	After five days	Reference range
White blood cells	28.4 × 10^9^/L	4.7 × 10^9^/L	4-10 × 10^9^/L
Hemoglobin	8.5 g/dL	12.2 g/dL	12-15 g/dL
Mean corpuscular volume	82 fL	82.7 fL	81-99 fL
Platelet	244 × 10^9^	201 × 10^9^	130-400 × 10^9^
Glucose	21 mmol/L	9.1 mmol/L	3.9-7.8 mmol/L
Blood urea nitrogen	7.6 mmol/L	3.7 mmol/L	3.2-7.1 mmol/L
Creatinine	72 mmol/L	55 mmol/L	46-110 mmol/L
Sodium	131 mmol/L	138 mmol/L	133-148 mmol/L
Potassium	5.2 mmol/L	3.6 mmol/L	3.4-5.1 mmol/L
Aspartate aminotransferase	78 U/L	24 U/L	15-46 U/L
Alanine aminotransferase	51 U/L	26.4 U/L	30-65 U/L
Total protein	88 g/dL	69 g/dL	63-82 g/dL
Albumin	49 g/dL	35 g/dL	30-50 g/dL
Alkaline phosphatase	91 U/L	43 U/L	38-126 U/L
Blood culture	Negative	Negative	Negative

**Table 5:** Summary of hemoglobin (Hb) electrophoresis

Hb type	Value	Reference range
Hb A	97.2%	96.5%-98%
Hb A2	2%	1.5%-3.5%
Hb F	0.8%	0%-1%

**Table 6:** Peripheral blood film

Blood film	
Red blood cells	Moderate to marked anisopoikilocytosis, polychromasia, nucleated cells, and moderate to marked blister cells; there are some of elliptocytes with severe hemolytic markers
White blood cells	Neutrophilic leukocytosis with left shift
Platelet	Normal size and shape

Taking the high prevalence of G6PD in our community and the clinical presentation into consideration, the diagnosis of G6PD, which is precipitated by DKA, was highly suspected. With the clinical impression of G6PD manifesting as acute hemolytic anemia and methemoglobinemia on the background of DKA, treatment was initiated. However, we cannot confirm our diagnosis at the time of admission, as the patient was in severe hemolysis. The patient had received four units of packed red blood cell transfusion, empirical antibiotics, and management in accordance with the hospital's diabetic ketoacidosis policy. He was given 1 g of ascorbic acid orally every day for three days with the aim of managing his methemoglobinemia. The patient was monitored with serial ABG, electrolytes, and CBC. His laboratory results ultimately demonstrated an alleviation of his hyperglycemia, metabolic acidosis, and an adequate decline in methemoglobin levels. After five days, the patient was discharged with improved hemolytic parameters, a methemoglobin level of 1%, and a 12.2 g/dL hemoglobin level, as demonstrated in Table 7. The diagnosis of G6PD was confirmed two months following the discharge with a G6PD level of 0.1 U/g.

**Table 7:** Hemolytic workup G6PD level taken after patient discharge in OPD to confirm the deficiency G6PD: glucose-6-phosphate dehydrogenase

Test result	Initial	After five days	Reference range
Reticulocyte	6%	1.2%	0%-2%
Lactate dehydrogenase	2254 U/L	625 U/L	100-200 U/L
Total bilirubin	131.6 μmol/L	14 μmol/L	3-22 μmol/L
Direct bilirubin	25.8 μmol/L	4.6 μmol/L	0-5 μmol/L
Coombs test, direct	Negative	-	Negative
Coombs test, indirect	Negative	-	Negative
G6PD	0.1 U/g		5.0-16.4 U/g
Vitamin B12 level	450 pg/mL	-	160-950 pg/mL

## Discussion

The relationship between diabetic ketoacidosis (DKA), glucose-6-phosphate dehydrogenase (G6PD) deficiency, and methemoglobinemia is complex and multifaceted. This case report illustrates a rare and significant interplay between these disorders, raising important considerations for clinical practice, particularly in patients not previously known to have G6PD deficiency.

G6PD deficiency is an inherited enzymatic disorder that predisposes individuals to hemolytic anemia, particularly when exposed to oxidative stressors such as infections, certain drugs, and foods [1]. In this case, the patient, who was not previously diagnosed with G6PD deficiency, presented with DKA, characterized by hyperglycemia, ketosis, and acidosis. Hyperglycemia and subsequent oxidative stress in DKA can impair the function of the G6PD enzyme, leading to the increased vulnerability of red blood cells (RBCs) to oxidative damage and subsequent hemolysis [6]. However, there is a study conducted on the Mediterranean variant of G6PD that found that DKA did not precipitate hemolysis; multiple case reports have documented significant hemolysis in non-Mediterranean variants of G6PD deficiency triggered by DKA, though the exact mechanisms remain elusive [9-14]. This indicates a potential variation in how different G6PD variants respond to oxidative stress, warranting further investigation into the genetic basis of these differences. These reports emphasize the need for clinicians to be vigilant for G6PD deficiency in patients presenting with DKA and hemolysis, especially in regions with a high prevalence of non-Mediterranean variants of G6PD deficiency. Although metformin was reported to trigger hemolysis in G6PD [15], our patient was already off medication for the last eight months prior to his admission.

Methemoglobinemia, a condition where hemoglobin is oxidized to methemoglobin, which cannot effectively release oxygen to tissues, can complicate cases of severe hemolytic anemia, particularly in G6PD-deficient patients [8]. This case presented an interesting scenario where a patient with undiagnosed G6PD deficiency and DKA also developed methemoglobinemia. This is notable as methemoglobinemia in G6PD deficiency can be precipitated by the increased oxidative stress during hemolytic episodes [8,16]. The therapeutic approach to methemoglobinemia in the context of G6PD deficiency poses a unique challenge. Methylene blue, a common treatment for methemoglobinemia, is contraindicated in G6PD-deficient individuals as it can exacerbate hemolysis [17-19]. In this case, the patient was treated with ascorbic acid, an antioxidant that aids in the reduction of methemoglobin to hemoglobin without inducing hemolysis, showcasing an effective management strategy for this complex condition [18]. The use of ascorbic acid versus methylene blue underscores the need for alternative therapeutic strategies in patients with G6PD deficiency and methemoglobinemia. Comparatively, other case reports have also indicated similar clinical presentations and management strategies in G6PD-deficient patients with DKA and methemoglobinemia, suggesting a pattern that clinicians should be aware of [10-14]. These reports emphasize the importance of prompt diagnosis and the use of appropriate antioxidant therapies to manage oxidative stress and hemolysis effectively [8,18].

This case underscores the importance of considering G6PD deficiency in patients presenting with DKA and unexplained hemolysis, particularly in populations with a high prevalence of G6PD deficiency [3]. It also highlights the need for the careful management of methemoglobinemia in such patients, avoiding treatments such as methylene blue that could worsen hemolysis [17,19]. In comparison, various reports have highlighted the need for awareness of potential complications arising from the interplay of these conditions [16,18]. For instance, in areas where G6PD deficiency is prevalent, such as Saudi Arabia, clinicians should be vigilant when managing DKA to prevent unrecognized hemolysis [3]. The recognition of the potential link between these conditions is crucial for timely diagnosis and appropriate treatment.

## Conclusions

This case report underscores the complex relationship between diabetic ketoacidosis (DKA), glucose-6-phosphate dehydrogenase (G6PD) deficiency, and methemoglobinemia. The successful management of the patient highlights the necessity of considering G6PD deficiency in patients with DKA who present with hemolysis and methemoglobinemia. The increased awareness and understanding of this association are crucial for prompt diagnosis and appropriate treatment, particularly in regions with a high prevalence of G6PD deficiency. Further research should focus on elucidating the biochemical and physiological mechanisms that link G6PD deficiency, DKA, and methemoglobinemia. Understanding these mechanisms can provide insights into why these conditions co-occur and inform targeted interventions.
